# Hierarchical ZnO Nanowires-loaded Sb-doped SnO_2_-ZnO Micrograting Pattern via Direct Imprinting-assisted Hydrothermal Growth and Its Selective Detection of Acetone Molecules

**DOI:** 10.1038/srep18731

**Published:** 2016-01-08

**Authors:** Hak-Jong Choi, Seon-Jin Choi, Soyoung Choo, Il-Doo Kim, Heon Lee

**Affiliations:** 1 Department of Materials Science and Engineering, Korea University, Anam-ro 145, Seongbuk-gu, Seoul 136-713, Republic of Korea; 2 Department of Materials Science and Engineering, Korea Advanced Institute of Science and Technology, 291 Daehak-ro, Yuseong-gu, Daejeon 305-701, Republic of Korea

## Abstract

We propose a novel synthetic route by combining imprinting transfer of a Sb-doped SnO_2_ (ATO)-ZnO composite micrograting pattern (MP), i.e., microstrip lines, on a sensor substrate and subsequent hydrothermal growth of ZnO nanowires (NWs) for producing a hierarchical ZnO NW-loaded ATO-ZnO MP as an improved chemo-resistive sensing layer. Here, ATO-ZnO MP structure with 3-μm line width, 9-μm pitch, and 6-μm height was fabricated by direct transfer of mixed ATO and ZnO nanoparticle (NP)-dispersed resists, which are pre-patterned on a polydimethylsiloxane (PDMS) mold. ZnO NWs with an average diameter of less than 50 nm and a height of 250 nm were quasi-vertically grown on the ATO-ZnO MP, leading to markedly enhanced surface area and heterojunction composites between each ATO NP, ZnO NP, and ZnO NW. A ZnO NW-loaded MP sensor with a relative ratio of 1:9 between ATO and ZnO (1:9 ATO-ZnO), exhibited highly sensitive and selective acetone sensing performance with 2.84-fold higher response (*R*_air_/*R*_gas_ = 12.8) compared to that (*R*_air_/*R*_gas_ = 4.5) of pristine 1:9 ATO-ZnO MP sensor at 5 ppm. Our results demonstrate the processing advantages of direct imprinting-assisted hydrothermal growth for large-scale homogeneous coating of hierarchical oxide layers, particularly for applications in highly sensitive and selective chemical sensors.

Human breath contains a number of biomarker molecules that are closely related to the physical condition of human. Recently, breath analysis has received intensive attention as a potential diagnostic method because it possesses several critical advantages such as non-invasiveness, simplicity of usage, and inexpensiveness. For example, higher acetone concentrations (900 ppb–1.2 ppm) have been observed in the breath of diabetes patients compared to the acetone concentration (300–600 ppb) of healthy human breath^1^. Moreover, it has been reported that higher concentrations of toluene and hydrogen sulfide in breath have strong correlations with the incidence of lung cancer and halitosis^2^,^3^. Thus far, several breath-analysis techniques have been introduced to detect biomarker molecules precisely for the non-invasive diagnosis of several diseases. For example, gas chromatography and mass spectroscopy (GC-MS) is a typical gas-analysis technique with high precision. However, analysis using GC-MS has several disadvantages such as high cost, difficulty of usage (requires a trained technician), and lengthy analysis time.

Semiconductor-metal-oxide (SMO)-based chemo-resistive sensors are receiving increasing attention for monitoring a person’s physical condition as well as for the diagnosis of several diseases^4^,^5^,^6^. Currently, n-type SMOs, such as SnO_2_, WO_3_, and ZnO, are the most interesting sensing materials because of their highly reactive sensing properties^7^. The sensing mechanism of SMO-based sensing materials is based on the change in resistance depending on the gas environment. To achieve highly sensitive gas sensing materials, structural modulation by maximizing the surface area of the SMO sensing layer is the foremost criterion. For this purpose, a number of one dimensional (1D) nanostructures, such as Si nanowire^8^, SnO_2_ nanofibers^9^,^10^, ZnO nanotubes^11^, and WO_3_ hemitubes^12^,^13^, have been synthesized for highly sensitive breath sensors, thereby demonstrating their potential application in the diagnosis of diseases. Moreover, enhanced sensing performance can be achieved by further increasing surface reaction sites by forming hierarchical structures of 1D-1D WO_3_ nanofibers^14^, 1D-0D urchin-like ZnO hollow hemispheres^15^, 3D α-Fe_2_O_3_ nanostructures^16^, and 3D flower-like ZnO structures^17^. In addition to the structural modulation, compositional modulation by forming hetero-junction composites between metal oxides can improve the sensing performance by adequately controlling the thickness of surface depletion layers^18^. Among various metal oxide composite sensing materials, SnO_2_-ZnO is a well-known metal-oxide-composite-sensing layer exhibiting high gas responses toward acetone^19^,^20^ and ethanol^21^,^22^,^23^.

In this work, we successfully fabricated a hierarchical ZnO nanowire-loaded Sb-doped SnO_2_-ZnO micrograting pattern (hereafter, ZnO NW-loaded ATO-ZnO MP) by simply combining direct imprinting^24^ of supporting ATO-ZnO MP structure and subsequent hydrothermal growth^25^ of ZnO NWs, which were used as a chemo-resistive sensor for the first time. First, direct imprinting, a kind of imprint lithography, was used to fabricate ZnO MPs due to following advantages. Imprint lithography is well-known as one of the next-generation lithographic techniques, and it is a simple, cost-effective, large-area, and high-resolution lithographic process^26^,^27^,^28^. In the case of the direct imprinting recently developed as a modified process, the additional advantages, such as reduction of the process steps and time and uniform formation of the pattern with various sizes and shapes on flat, curved, and patterned surfaces composed of various materials, can be acquired because functional patterns are directly formed using inorganic nanoparticles (NPs)^29^, sol-gel solution^30^, and spin-on-glass^31^. We synthesized the ZnO NW–loaded pure ZnO MP structure, which helps to increase the surface area, thereby improving the response of the sensor to analytes by forming a hierarchical 1D NW-1D strip line structure with homogeneous junctions. In addition, ATO-ZnO composite MP structure fabricated by using mixed ATO and ZnO NPs was achieved for further enhancing the gas sensing performance. We compared the sensing characteristics between different compositional ratios of ATO and ZnO. The direct imprinting technique combined with hydrothermal growth generates a hierarchical ZnO NW-loaded ATO-ZnO MP structure that provides highly sensitive and selective acetone detection performance.

## Result

### Synthesis of hierarchical ZnO NW-loaded ATO-ZnO MP

**Figure 1:**
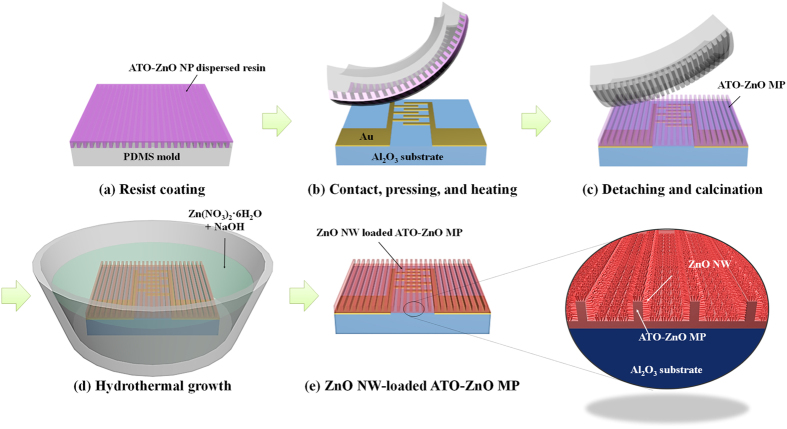
Schematic illustration for the fabrication process of ZnO NW-loaded ATO-ZnO MP based gas sensors. (**a**) Resist coating using ATO-ZnO NPs-dispersed resin on a PDMS mold, (**b**) Imprinting transfer of the resist on an Au-coated Al_2_O_3_ sensor substrate via contact, pressing, and heating step, (**c**) Calcination of transferred ATO-ZnO MP, (**d**) Hydrothermal growth of ZnO NWs on ATO-ZnO MP, (**e**) ZnO NW-loaded ATO-ZnO MP based gas sensor. The magnified image highlights the hierarchical structure of 1D NW-1D microstrip ATO-ZnO MP with enhanced surface area.

We fabricated hierarchical ZnO NW-loaded ATO-ZnO MP structures on Al_2_O_3_ sensor substrates using a hybrid process that combines direct imprinting and hydrothermal growth (Fig. 1). As sensing materials, ZnO and ATO-ZnO were used for preparing ZnO NW-loaded ZnO MP, and ZnO NW-loaded ATO-ZnO MP structures, respectively, through direct imprinting with 3-μm line width, 9-μm pitch, and 6-μm height. The direct imprinting resists consist of dispersed NPs of ATO and ZnO. The resists were mixed in appropriate weight ratios to form MPs of six different compositional ratios between ATO and ZnO, i.e., 0:10 (pristine ZnO), 1:9, 3:7, 5:5, 7:3, and 9:1. First, each resist consisting of dispersed ZnO NPs or ATO-ZnO composite NPs were spin-casted on the PDMS mold at 1500 rpm for 30 s (Fig. 1a). Then, the conformal contact between an interdigitated Au-coated Al_2_O_3_ sensor substrate and the resist-coated PDMS mold was performed followed by pressurization and subsequent annealing step (Fig. 1b). During the heating process, the ZnO and ATO-ZnO NP based resists were thermally cured. Then, PDMS mold was detached from the sensor substrate, leaving each ZnO MP and ATO-ZnO MP structure. Finally, the ZnO MP and ATO-ZnO MP sensor were calcined at 500 °C for 1 h (Fig. 1c). Next, ZnO NWs were hydrothermally grown on pristine ZnO MP and ATO-ZnO composite MP supporting structure, resulting in the formation of ZnO NW-loaded ZnO MP and ZnO NW-loaded ATO-ZnO MP sensors (Fig. 1d,e).

**Figure 2:**
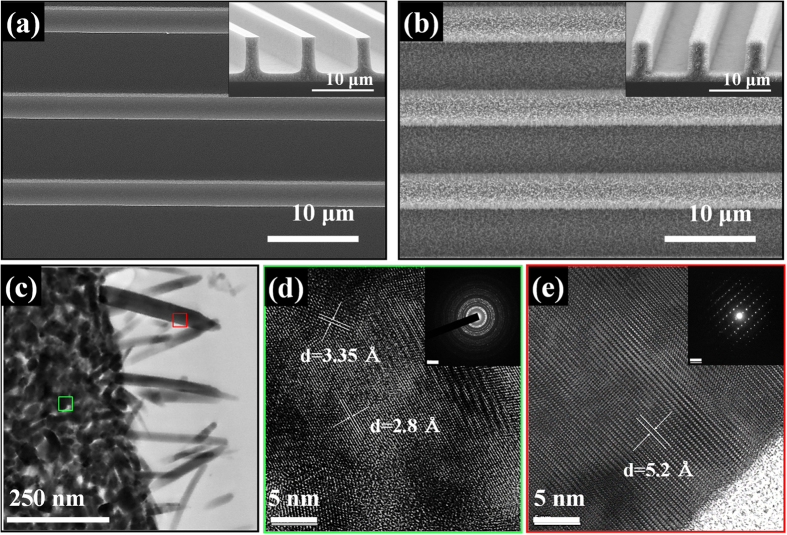
SEM micrograph: (a) 1:9 ATO-ZnO MP and (b) Hierarchical ZnO NW-loaded 1:9 ATO-ZnO MP. (**c**) TEM micrograph of ZnO NW-loaded 1:9 ATO-ZnO MP, (**d**) Magnified HR-TEM image from green box in single strip line of MP in Figure 2c (inset: ring pattern), (**e**) HR-TEM image of ZnO NW from red box in Figure 2c (inset: SAED pattern).

Structural and morphological observations of hierarchical ZnO NW-loaded ATO-ZnO MP structures were performed using FE-SEM and FE-TEM. Figure 2a shows the SEM micrograph of the ATO-ZnO MP with a pitch of 9 μm, height of 6 μm, and line-width of 3 μm, and a residual layer with a thickness of 1 μm. The hierarchical ZnO NW-loaded ATO-ZnO MP was successfully obtained after subsequent hydrothermal growth of ZnO by forming ZnO NWs on an ATO-ZnO MP (Fig. 2b). The fabricated ATO-ZnO MP and hierarchical structure of ZnO NW-loaded ATO-ZnO MP exhibited a well-defined structure after the processes, as investigated in the tilted SEM images (in the inset of Fig. 2a,b). In order to investigate a cross-sectional view of the ZnO NW-loaded ATO-ZnO MP, a TEM micrograph was obtained at the interface of ATO-ZnO MP and ZnO NWs (Fig. 2c). It was revealed that ZnO NWs, which have an average diameter of less than 50 nm and height of 250 nm, were quasi-vertically grown on the ATO-ZnO MP. High-resolution TEM (HR-TEM) observation was performed to investigate the crystal structures of the individual ZnO NW and ATO-ZnO MP, respectively. As shown in Fig. 2d, the ATO-ZnO MP presents two different interplanar distances of 3.35 and 2.8 Å, which correspond to the Cassiterite structure of SnO_2_ with the (110) crystallographic plane and the wurtzite structure of ZnO with the (100) crystallographic plane. In addition, an HR-TEM micrograph for some of the ZnO NWs shows the interplanar distance of 5.2 Å, corresponding to the (001) crystallographic plane with single crystallinity (Fig. 2e). The selected area electron diffraction (SAED) patterns in insets of Fig. 1d,e reveal the polycrystalline structure of the ATO-ZnO MP exhibiting crystal planes, such as (100), (101), and (002) for ZnO, and (110), (101), and (301) for SnO_2_, respectively. In addition, the single crystalline structure of the ZnO NWs presenting the crystal plane of (001) was clearly observed. Elemental mapping images of Fig. 2c for Zn, O, Sn, and Sb atoms were acquired using EDX (Supporting Information Figure S1) to investigate the compositional distribution of ZnO NW-loaded ATO-ZnO MP. The result revealed that Zn and O atoms were well-distributed in ZnO NW-loaded 1:9 ATO-ZnO MP, whereas Sn and Sb atoms were sparsely distributed in ATO-ZnO MP.

### Microstructure and Chemical Analysis of hierarchical ZnO NW-loaded ATO-ZnO MP

**Figure 3:**
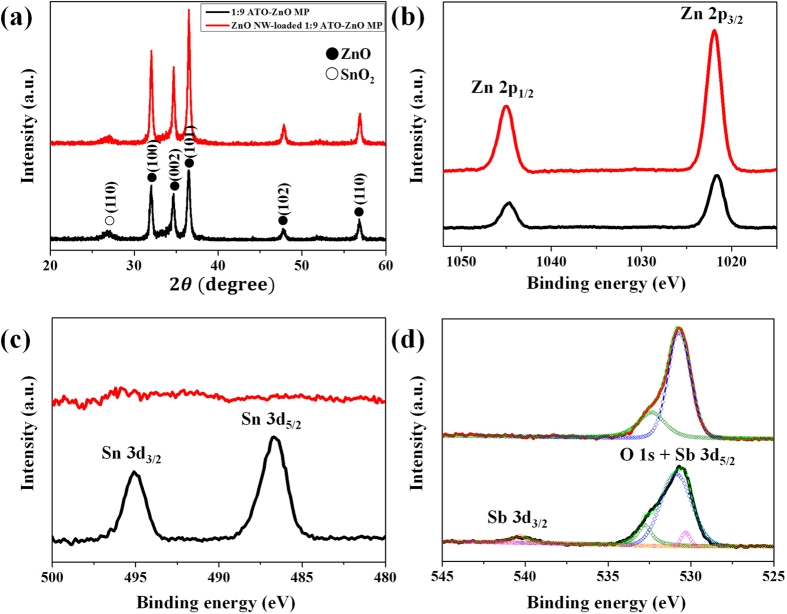
(**a**) X-ray diffraction pattern and XPS spectra of (**b**) Zn 2p, (**c**) Sn 3d, and (**d**) O 1s + Sb 3d for 1:9 ATO-ZnO MP and hierarchical ZnO NW-loaded 1:9 ATO-ZnO MP.

Crystallographic structure, chemical composition, and bonding states were examined with the ATO-ZnO MP and ZnO NW-loaded 1:9 ATO-ZnO MP using an X-ray diffraction (XRD) goniometer and X-ray photoelectron spectroscopy (XPS) (Fig. 3). The XRD diffraction peak at Bragg angles (2*θ*) for ATO-ZnO MPs and ZnO NW-loaded ATO-ZnO MP were presented at 26.9°, 32.2°, 34.5°, 36.3°, 47.5°, and 56.6°, which correspond to the (110) plane for Cassiterite SnO_2_ structure and the (100), (002), (101), (102), and (110) plane for Wurtzite ZnO structure (Fig. 3a). The diffraction peaks for ATO were slightly shifted compared to the peaks of pristine SnO_2_ reported in the literature because of the doping of Sb atoms^32^. Moreover, the relative peak intensity of ZnO compared to that of SnO_2_ was higher for the hierarchical ZnO NW-loaded ATO-ZnO MP than for the ATO-ZnO MP because of the addition of ZnO NWs. The surface compositions of ATO-ZnO MP and ZnO NW-loaded ATO-ZnO MP were also surveyed by measuring XPS (Supporting Information Figure S2). High-resolution XPS analysis was performed for Zn, Sn, O, and Sb atoms (Fig. 3b–d). Figure 3b shows the high-resolution XPS spectra in the range of the Zn 2*p* orbital peak, where two distinct peaks at binding energies of 1021.6 and 1044.6 eV corresponding to the 2*p*_3/2_ and 2*p*_1/2_ states of Zn^2+^ can be observed. The peak intensity of the Zn 2*p* peaks increased after the hydrothermal growth of ZnO NWs, which formed a dense ZnO NW forest on the ATO-ZnO MP. In contrast to the high-resolution XPS spectra of the Zn atom, XPS peaks of the Sn 3*d* orbital were observed only in the ATO-ZnO MP, with binding energies of 486.6 and 495.1 eV for Sn 3*d*_5/2_ and 3*d*_3/2_, respectively (Fig. 3c). This implies that a dense ZnO NW forest fully covered the surface of the ATO-ZnO MP. Figure 3d shows O 1*s* (blue, green) and Sb 3*d* (magenta, orange) spectra for the ATO-ZnO MP and ZnO NW-loaded ATO-ZnO MP. The Sb 3*d* spectrum was observed only in the ATO-ZnO MP, as in the analysis of the Sn spectrum, for the same reason. In the ATO-ZnO MP, the asymmetric O 1*s* peak overlapped with the Sb 3*d*_5/2_ peak, which has a peak value of 530.7 eV. The overlapping peak can be resolved into several peaks that correspond to binding energies of 530.3 (Sb 3*d*_5/2_), 530.9 (O 1*s*), and 532.8 eV (O 1*s*), and the Sb 3*d*_3/2_ peak is obtained with a binding energy of 540.4 eV. The binding energies of 530.3 eV and 540.4 eV for Sb 3*d* are characteristic of the Sb^5+^ in Sb_2_O_5_. Furthermore, the binding energies of 530.9 eV and 532.8 eV represent the bonding states of oxygen; they correspond to the O^2−^ in the metal oxide and the surface adsorbed oxygen species such as OH^−^, respectively.

### Gas-sensing Characteristics

**Figure 4:**
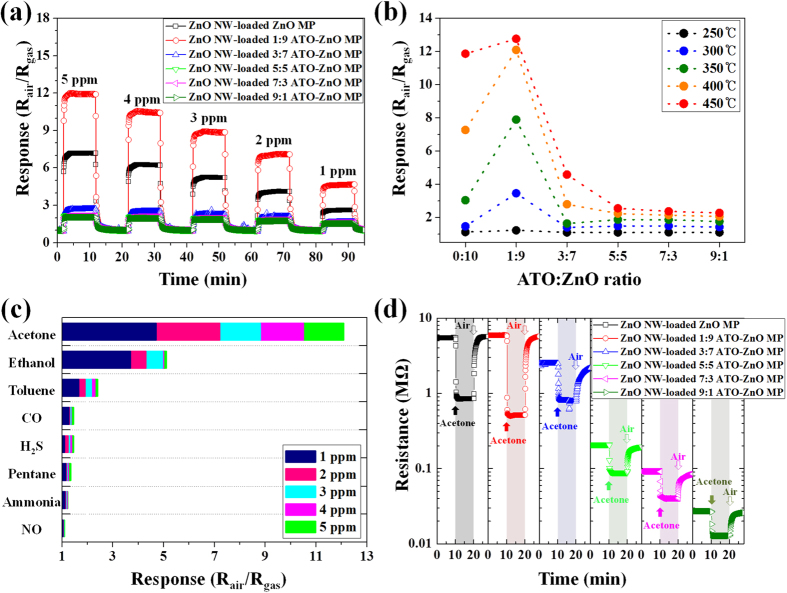
(**a**) Dynamic acetone sensing characteristic of ZnO NW-loaded ZnO MP and ZnO NW-loaded ATO-ZnO MP sensors with five different compositions at 400 °C in a concentration range of 1–5 ppm, (**b**) Temperature-dependent acetone sensing characteristics of the sensors at 5 ppm in a temperature range of 250–450 °C, (**c**) Selective sensing characteristics of ZnO NW-loaded 1:9 ATO-ZnO MP sensor in a concentration range of 1–5 ppm at 400 °C toward acetone and other interfering gases, and (**d**) Dynamic resistance transition characteristic of the sensors toward 5 ppm of acetone at 400 °C.

Gas-sensing characteristics were investigated using ZnO NW-loaded ATO-ZnO MP with six different compositions (0:10, 1:9, 3:7, 5:5, 7:3, 9:1) toward multiple analytes including acetone, H_2_S, and toluene, which are respectively known as biomarkers of diabetes, halitosis, and lung cancer in exhaled breath, at different operating temperature in highly humid ambient (90% RH). Figure 4a shows the dynamic acetone response characteristics of ZnO NW-loaded ATO-ZnO MP sensors, investigated at 400 °C in the concentration range of 1–5 ppm. Among six sensor specimens, ZnO NW-loaded ZnO MP and ZnO NW-loaded 1:9 ATO-ZnO MP showed remarkably increased acetone response, compared with ZnO MP and ATO-ZnO MP (Supporting Information Figure S3). In the case of a ZnO NW-loaded ZnO MP sensor, the acetone response value was increased up to 7.3 at 5 ppm and maintained 2.6 at 1 ppm. Interestingly, the acetone detection capability was significantly enhanced by the addition of a small amount (10 wt%) of ATO. Over a 1.5-fold improved acetone sensing property was achieved with ZnO NW-loaded 1:9 ATO-ZnO MP sensor, which exhibited the response of 12.1 at 5 ppm acetone as compared to the response (*R*_*air*_/*R*_*gas*_ = 7.3 at 5 ppm) of ZnO NW-loaded ZnO MP sensor. However, further increments of the ATO ratio in ATO-ZnO composite MP structure, i.e., 3:7, 5:5, 7:3, and 9:1 in the relative ratio between ATO and ZnO, did not greatly influence the improvement of the acetone sensing performances, which have response values below 3 in a concentration range of 1–5 ppm. The dynamic H_2_S and toluene response characteristics were also investigated for different hierarchical ZnO NW-loaded ATO-ZnO MP sensors at 400 °C in the same concentration range (Supporting Information Figure S4). The result revealed that all hierarchical ZnO NW-loaded ATO-ZnO MP sensors with different compositional ratios between ATO and ZnO exhibited minor responses (R_*air*_/R_*gas*_ < 2) for H_2_S and (R_*air*_/R_*gas*_ ~ 3) for toluene, respectively. It should be noted that the H_2_S sensing properties were degraded by the addition of ATO in the hierarchical structure sensors while improved acetone sensing properties were achieved for ZnO NW-loaded 1:9 ATO-ZnO MP sensor, leading to superior acetone selectivity toward H_2_S. The temperature-dependent acetone sensing characteristics of hierarchical ZnO NW-loaded ATO-ZnO MP sensors at 5 ppm in a temperature range of 250–450 °C were investigated (Fig. 4b). The result indicated that ZnO NW-loaded 1:9 ATO-ZnO MP sensor exhibited the optimum composition with the highest response of 12.8 at 450 °C. The responses of ZnO NW-loaded 1:9 ATO-ZnO MP sensor were steadily increased with an increase in the operating temperature from 250 °C to 450 °C. In contrast, the ZnO NW-loaded ATO-ZnO MP sensors with compositions of 3:7, 5:5, 7:3, and 9:1 showed no significant improvement in acetone response from 250 °C to 450 °C. For a pristine ZnO NW-loaded ZnO MP sensor, a noticeable increment in acetone responses of 7.3 and 11.8 was observed at high temperatures of 400 °C and 450 °C, respectively. The temperature-dependent H_2_S and toluene response characteristics of six different hierarchical structure sensors at 5 ppm was also evaluated at 250–450 °C (Supporting Information Figure S5). Selective acetone detection properties were investigated, which can be potentially applied for the diagnosis of diabetes by analyzing breath components. Figure 4c shows the selective sensing characteristics of ZnO NW-loaded 1:9 ATO-ZnO MP sensor in a concentration range of 1–5 ppm at 400 °C towards acetone and other interfering gases. The result demonstrate that a highly acetone-selective characteristic (*R*_*air*_*/R*_*gas*_ = 12.1 at 5 ppm) was obtained using a ZnO NW-loaded 1:9 ATO-ZnO MP sensor with a observable response (*R*_*air*_*/R*_*gas*_ = 5.1 at 5 ppm) toward ethanol and negligible responses (*R*_*air*_*/R*_*gas*_ < 3 at 5 ppm) to interfering analytes such as toluene, CO, H_2_S, pentane, ammonia, and NO.

Table 1 is the summary of the recent publications related to the pristine ZnO as well as Sb-doped ZnO composites for acetone sensing layers. The previous studies have demonstrated the improved acetone sensing performance with Sb dopant loaded ZnO composites. However, most of the previous studies were performed in relatively dry ambient. Considering the degrading sensing characteristic in highly humid ambient due to the adsorption of water molecules on the surface as well as the capillary water condensation within the small pores^33^, the present work demonstrated highly sensitive acetone sensing in humid ambient even at low ppm level, which shows high potential for application in breath acetone analysis. Moreover, to the best of our knowledge, Sb-doped SnO_2_ loaded ZnO nanostructures have never been investigated.

## Discussion

To understand the sensing mechanism, the dynamic resistance transition characteristic was examined for six different hierarchical structure sensors at an operating temperature of 400 °C (Fig. 4d). The results showed a dramatic decrease in baseline resistance with an increase in ATO ratio. The variation of resistance can be described based on following proposed mechanism (Supporting Information Figure S6). Generally, ATO is well-known as one of the transparent conducting oxides that have very low resistance^34^. For a ZnO NW-loaded 1:9 ATO-ZnO MP sensor, the baseline resistance was maintained as 5.88 MΩ, which is compatible with the pristine ZnO NW-loaded ZnO sensor (5.42 MΩ), because most of randomly-distributed ATO (10 wt%) NPs are isolated by ZnO NPs due to a small loading amount of ATO. On the other hand, as the ATO ratio is further increased from 3:7 to 9:1, the baseline resistances were gradually decreased from 2.49 MΩ to 27.17 kΩ, which was attributed to the dominant current flow through ATO NPs or agglomerated ATO NPs. The improved acetone response behavior of the ZnO NW-loaded 1:9 ATO-ZnO MP sensor can be explained in terms of structural and compositional aspects. For the structural aspect, increased surface area of hierarchical structure of ATO-ZnO MP by forming a ZnO NW forest can generate an enhanced acetone response due to the remarkably increased surface reaction sites. Basically, SnO_2_ and ZnO are well-known n-type semiconductor metal oxides. When these materials are exposed in baseline air ambient, chemisorbed oxygen species such as O_2_^–^, O^–^, and O^2–^ can be formed on the surface by trapping electrons from the conduction band. When reducing analytes such as acetone are exposed to the n-type sensors, chemisorbed oxygen species can react with acetone by donating electrons back to the conduction band with the following chemical reactions^35^:
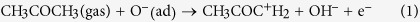

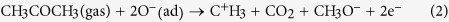


**Figure 5:**
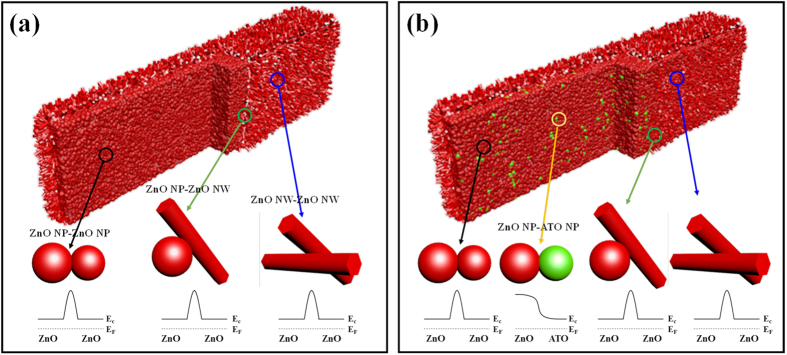
Schematic diagram illustrating contact potential barriers formed at interfaces among ZnO NP, ATO NP, and ZnO NW for (**a**) ZnO NW-loaded ZnO MP and (**b**) ZnO NW-loaded 1:9 ATO-ZnO MP.

Therefore, increased surface reaction between chemisorbed oxygen species and acetone can be one reason for the improved acetone sensing performance. From the compositional aspect, hetero-junction created at the interfaces between ATO and ZnO domains greatly influences the improved acetone sensing performance. The hetero-structures between SnO_2_ and ZnO are known to enhance the gas sensitivity and selectivity^36^,^37^,^38^. Figure 5a shows a schematic diagram illustrating the formation of possible depletion layers for pristine ZnO NW-loaded ZnO MP. For ZnO NW-loaded ZnO MP sensor, three different kinds of potential barriers with homo-junction can be formed at the interface of ZnO NP-ZnO NP, ZnO NW-ZnO NP, and ZnO NW-ZnO NW. In the case of a ZnO NW-loaded ATO-ZnO MP sensor, hetero-junction potential barriers are created due to work function difference between SnO_2_ and ZnO, as shown in Figure 5b. When SnO_2_ and ZnO form a hetero-junction, electrons will flow from SnO_2_ with a lower work function (φ = 4.9 eV)^39^ to ZnO with a higher work function (φ = 5.2 eV)^40^ until the Fermi levels of SnO_2_ and ZnO are equalized by forming a depletion layer. Then, an additional depletion layer can also exist at the hetero-junction interface of the ZnO NP-ATO NP within the ATO-ZnO MP structure. The depletion layer plays an important role in the modulation of carrier transport, which is attributed to improving acetone sensitivity and selectivity as compared to the pristine ZnO NW-loaded ZnO MP sensor. Another important parameter related with enhanced sensing performance is Sb-doping as an additive in the SnO_2_ matrix. The doping of Sb_2_O_5_ as an oxidized form of Sb in SnO_2_ base material is frequently attempted in order to improve the selective gas detection by increasing the response to target analytes such as ethanol^41^ and isobutane^42^ and by reducing the response to interfering analytes such as H_2_^43^. In the present study, Sb in SnO_2_ NPs was found to exist as Sb_2_O_5_ according to the XPS analysis (Fig. 3d). Most of the additive Sb^5+^ ions in Sb_2_O_5_ probably act as n-type dopant through a substitution of Sn^4+^, as described in the following reaction^44^.
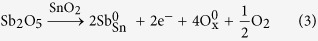


The reaction can contribute to the improved acetone sensing performance by donating electrons to the ZnO NW-loaded ATO-ZnO MP sensor, which can induce more chemisorbed oxygen species on the surface^44^. In addition, additive Sb^5+^ can reduce the work function of SnO_2_ to less than 4.9 eV while increasing the depletion region^45^. Therefore, hetero-junctions formed between ATO and ZnO can further improve acetone sensing performance. Moreover, selective sensing properties of the pristine ZnO NW-loaded ZnO MP exhibited high responses toward all the interfering analytes, which implies no selectivity to acetone (Figure S7). The result confirmed that the addition of a small amount of ATO dopants can induce enhancement in acetone selective property by effectively forming surface depletion layers. Even though the depletion layers were formed between the hetero-junctions, the base resistance of ZnO NW-loaded 1:9 ATO-ZnO MP sensor was similar to the pristine ZnO NW-loaded ZnO MP sensor (Fig. 4d), which was mainly attributed to the small amount of ATO addition.

**Figure 6:**
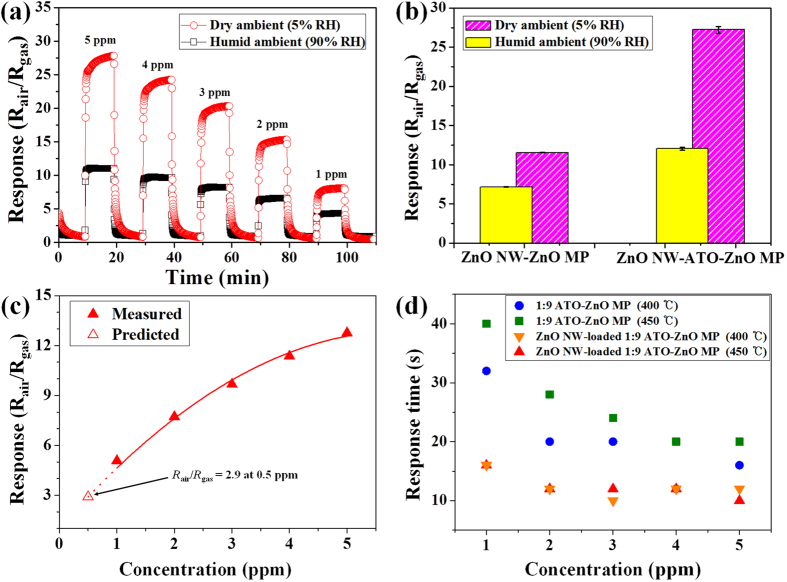
(**a**) Dynamic response transition of ZnO NW-loaded 1:9 ATO-ZnO MP sensor in dry and humid ambient at 400 °C. (**b**) Response characteristics of pristine ZnO NW-loaded ZnO MP sensor and ZnO NW-loaded 1:9 ATO-ZnO MP sensor to 5 ppm of acetone in dry and humid ambient at 400 °C. (**c**) Detection limit of a ZnO NW-loaded 1:9 ATO-ZnO MP sensor measured at 450 °C. (**d**) Response time characteristics of 1:9 ATO-ZnO MP and ZnO NW-loaded 1:9 ATO-ZnO MP sensors measured at 400 °C and 450 °C in a concentration range of 1–5 ppm.

The effect of humidity levels on acetone sensing properties was investigated using the pristine ZnO NW-loaded ZnO MP sensor and ZnO NW-loaded 1:9 ATO-ZnO MP sensor (Fig. 6a,b). The dynamic response characteristics of ZnO NW-loaded 1:9 ATO-ZnO MP sensor showed higher response (R_*air*_/R_*gas*_ = 18.9 at 5 ppm) to acetone in dry ambient (5% RH) than that (R_*air*_/R_*gas*_ = 11.4 at 5 ppm) in humid ambient (90% RH) (Fig. 6a). The enhanced acetone sensitivity in dry ambient was identically observed with the pristine ZnO NW-loaded ZnO MP sensor as compared to the sensitivity in humid ambient (Fig. 6b). It should be noted that a comparable acetone sensing characteristic of ZnO NW-loaded 1:9 ATO-ZnO MP sensor in humid ambient was observed as compared to the response with the pristine ZnO NW-loaded ZnO MP sensor in dry ambient, which was mainly attributed to the doping effect of Sb-doped SnO_2_ (ATO). The decreased responses of the sensors in humid ambient were resulted from the formation of the hydroxyl group (–OH) on the surface instead of chemisorbed oxygen species, leading to less resistance changes to acetone^33^. This result indicates the critical role of the chemisorbed oxygen species on the surface of the sensors to improve the sensing performance.

The detection limit of the hierarchical ZnO NW-loaded 1:9 ATO-ZnO MP sensor toward acetone was investigated through quadratic plotting based on the measured response values at 450 °C in the concentration range of 1–5 ppm (Fig. 6c). The quadratic plotting indicates that the ZnO NW-loaded 1:9 ATO-ZnO MP sensor is capable of detecting 500 ppb of acetone with the response value of 2.9, which is comparable with the acetone concentration of the exhaled breath of a healthy human. To demonstrate the potential application for real-time breath analysis for the diagnosis of diabetes, the response time characteristics toward acetone were evaluated using the 1:9 ATO-ZnO MP and ZnO NW-loaded 1:9 ATO-ZnO MP sensors (Fig. 6d). It is important to note that the ZnO NW-loaded ATO-ZnO MP sensor showed a very fast response time (<16 s) in all concentration ranges of 1–5 ppm at 400 °C and 450 °C compared to that (<40 s) of the ATO-ZnO MP sensor. In addition, the response times of the ZnO NW-loaded ATO-ZnO MP sensor showed less variation when the concentration of acetone was decreased from 5 ppm to 1 ppm. In contrast, large variations in response times were observed with the ATO-ZnO MP sensor depending on the variation in acetone concentration, i.e., a dramatic increase in response time when acetone concentration dropped to 1 ppm.

In summary, we developed a facile and cost-effective fabrication process based on the hybridization of direct imprinting and hydrothermal growth for producing hierarchical ZnO NW-loaded ATO-ZnO MP structures, which are structurally and compositionally optimized for applications in highly sensitive and selective chemo-resistive sensors. Compared with an ATO-ZnO MP sensor, the gas response of the hierarchical ZnO NW-loaded ATO-ZnO MP sensor was dramatically enhanced for acetone by 2.8 times at 450 °C (*R*_air_/*R*_gas_ = 12.8 at 5 ppm) at high humidity condition (90% RH), whereas no significant change was observed for H_2_S and toluene, indicating superior cross-sensitivities against other interfering gases. In addition, the ZnO NW-loaded ATO-ZnO MP sensor showed a fast response time (<16 s) characteristic toward acetone in a concentration range of 1–5 ppm. Our results verified that the novel hybrid synthetic route by combining imprinting lithography and hydrothermal growth, which are used for the fabrication of ZnO NW-loaded ATO-ZnO MP structures, is highly suitable for applications in high-performance gas sensors with enhanced surface area. Further improvement in gas responses can be achieved by simple optimization in materials, additives, and compositions of hierarchical NWs-anchored micrograting pattern, enabling the practical application of large-scale imprinting lithography-based chemical sensors.

## Method

### Preparation of micrograting pattern (MP)

In order to fabricate the Sb-doped SnO_2_ (ATO)-ZnO micrograting pattern (MP), imprint resists were prepared using ATO and ZnO NPs, respectively. A resist consisting of ATO NPs (Ditto technology) of diameter less than 50 nm at a concentration of 15 wt% was prepared by dispensing ATO NPs into a mixture of dipentaerythritol hexaacrylate (DPHA, Aldrich), isopropanol (IPA, Aldrich), and tert-butyl peroxy-2-ethylhexanoate (Trigonox® 21S, AkzoNobel). Then, the resist composed of dispersed ATO NPs was homogenized by stirring at a rotation speed of 300 rpm at 25 °C for 2 h. The Sn/Sb molar ratio was 85:15. Similarly, a resist consisting of dispersed ZnO NPs was prepared using 40 wt% ZnO NPs in ethanol (Aldrich), DPHA, and Trigonox® 21S. The ATO and ZnO NP-based resists were then mixed together to produce ATO/ZnO composite with weight ratios of 1:9, 3:7, 5:5, 7:3, and 9:1. In order to achieve an MP structure, polydimethylsiloxane (PDMS) was used as an imprint mold due to its high gas permeability and low surface energy. First, Sylgard 184A and 184B (Dowhitech Silicon Co.) were mixed at a volumetric ratio of 10:1. Second, the mixture was poured onto a master mold with a microscale grating pattern (pitch = 9 μm, height = 6 μm, line-width = 3 μm), which was fabricated by using photolithography and reactive ion etching, and cured at 80 °C for 2 h. Finally, PDMS mold was peeled off from the master mold, resulting in the inverted patterns of PDMS mold.

### Fabrication of Hierarchical ZnO NW-loaded ATO-ZnO MP

In order to synthesize the hierarchical structure, ZnO NWs were hydrothermally grown on ZnO MP and ATO-ZnO composite MP structures. First, 1 mM of zinc nitrate hexahydrate [Zn(NO_3_)_2_·6H_2_O] and 90 mM of sodium hydroxide [NaOH] were dissolved in deionized (DI) water. Second, cleaned substrates with the MP were immersed in the solution. Subsequently, the solution was heated to 50 °C and maintained for 2 h with gentle stirring. After ZnO NWs growth, the substrates were cleaned with ethanol and DI water, followed by drying under N_2_ flow to obtain the completed ZnO NW-loaded ATO-ZnO MP on a sensor substrate.

### Structural and Compositional Characterization

The surface morphology of ATO-ZnO MP and ZnO NW-loaded ATO-ZnO MP was investigated using field-emission scanning electron microscopy (FE-SEM, Hitachi, S-4300). Field emission transmission electron microscopy (FE-TEM, JEOL, JEM-2100F) with energy dispersive X-ray (EDX) spectroscopy was used to analyze the crystallinity and atomic distribution of hierarchical ZnO NW-loaded ATO-ZnO MP. The TEM sample of the ZnO NW-loaded ATO-ZnO MP was prepared using a dual-beam focused ion beam (FIB, FEI Nova 600) method. The crystal structures of the ATO-ZnO MP and hierarchical ZnO NW-loaded ATO-ZnO MP were analyzed with X-ray diffraction (XRD, Rigaku D/MAX-2500V/PC) based on Cu Kα radiation (λ = 1.54 Å). The bonding state and chemical composition of the main elements were investigated using X-ray photoelectron spectroscopy (XPS, Sigma Probe, Thermo VG Scientific) with Al Kα radiation.

### Gas-sensing Characterization

The sensor substrate was prepared using an Al_2_O_3_ substrate (3 mm × 3 mm), which was patterned with interdigitated finger-type Au electrodes on the front side and a microheater at the back for a sensing test of a hierarchical ZnO NW-loaded ATO-ZnO MP. In order to characterize the gas-sensing performances, a specialized gas sensor testing system was used, as described elsewhere^46^. Before starting the test, all sensors were stabilized in the baseline air ambient at a relative humidity (RH) of 90% for 6 h, considering the environment of exhaled breath at an operating temperature. Then, analyte gases including acetone, H_2_S, and toluene, which are known as biomarkers of diabetes, halitosis, and lung cancer, respectively, were introduced to the sensors. The concentrations of these analytes were tuned between 1 and 5 ppm, mixed in the baseline air, by controlling the flow rates of the test gas and baseline air while keeping a constant flow rate of 1000 sccm. Cyclic exposures of 10 min to the test gas followed by 10 min in the baseline air were performed. The resistance changes were measured using a data-acquisition system (34972A, Agilent) with a 16-channel multiplexer (34902A, Agilent) for simultaneous measurement of multiple sensors. The measured resistances were converted into the *R*_air_/*R*_gas_ ratio (where *R*_air_ and *R*_gas_ are the sensor resistances in baseline air ambient and analyte gas ambient, respectively), which is defined as the sensor response. The operating temperatures of the sensors were controlled by applying DC voltage in a range of 3.6–6.4 V using a DC power supply (E3647A, Agilent).

## Additional Information

**How to cite this article**: Choi, H.-J. *et al.* Hierarchical ZnO Nanowires-loaded Sb-doped SnO_2_-ZnO Micrograting Pattern via Direct Imprinting-assisted Hydrothermal Growth and Its Selective Detection of Acetone Molecules. *Sci. Rep.* **6**, 18731; doi: 10.1038/srep18731 (2016).

## Supplementary Material

Supplementary Information

## Figures and Tables

**Table 1:** Recent publications of acetone sensors based on pristine ZnO and Sb-doped ZnO nanostructures.

Composites	Response (R_air_/R_gas_)	Detection limit	Testing ambient	Response time	Operating temperature	Ref.
ZnO nanoparticles	10.5 (@ 100 ppm)	~2 (@ 10 ppm)	20% RH	13 sec	300 °C	47
Hierarchical ZnO spheres	33 (@ 100 ppm)	1.16 (@ 25 ppb)	25% RH	3 sec	230 °C	48
Porous ZnO sphere	186 (@ 100 ppm)	19 (@ 2 ppm)	—	3–4 sec	310 °C	49
ZnO nanorods	30.4 (@ 100 ppm)	1.9 (@ 1 ppm)	Dry air	5 sec	300 °C	50
ZnO Nanosheets	37.5 (@ 100 ppm)	<15 (@ 10 ppm)	25% RH	10 sec	420 °C	51
Sb-ZnO nanoparticles	~32.5 (@ 100 ppm)	—	—	10 sec	370 °C	52
Sb-ZnO nanorods	95 (@ 100 ppm)	—	Dry air	5 sec	190 °C	53
ZnO NW-loaded ATO-ZnO MP	12.1 (@ 5 ppm)	4.3 (@ 1 ppm)	90% RH	<16 sec	400 °C	This work
	27.8 (@ 5 ppm)	8.1 (@ 1 ppm)	5% RH	32 sec		
