# Supplementary material for: Hierarchical ZnO Nanowires-loaded Sb-doped SnO2-ZnO Micrograting Pattern via Direct Imprinting-assisted Hydrothermal Growth and Its Selective Detection of Acetone Molecules
Source: Sci Rep. 2016 Jan 8;6:18731. doi: 10.1038/srep18731 (PMC4705460; doi:10.1038/srep18731)
Supplement: Supplementary Information [file srep18731-s1.doc]

Supporting Information

Hierarchical ZnO Nanowires-loaded Sb-doped SnO_2_-ZnO Micrograting Pattern via Direct Imprinting-assisted Hydrothermal Growth and Its Selective Detection of Acetone Molecules

Hak-Jong Choi,^1,3^ Seon-Jin Choi,^2,3^ Soyoung Choo,^1^ Il-Doo Kim,^2^,* and Heon Lee^1^,*

^1^ Department of Materials Science and Engineering, Korea University, Anam-ro 145, Seongbuk-gu, Seoul 136-713, Republic of Korea

^2^ Department of Materials Science and Engineering, Korea Advanced Institute of Science and Technology, 291 Daehak-ro, Yuseong-gu, Daejeon 305-701, Republic of Korea

^3^ These authors are equally contributed to this work

*Corresponding authors: E-mail: [heonlee@korea.ac.kr](mailto:heonlee@korea.ac.kr)

E-mail: [idkim@kaist.ac.kr](mailto:idkim@kaist.ac.kr)


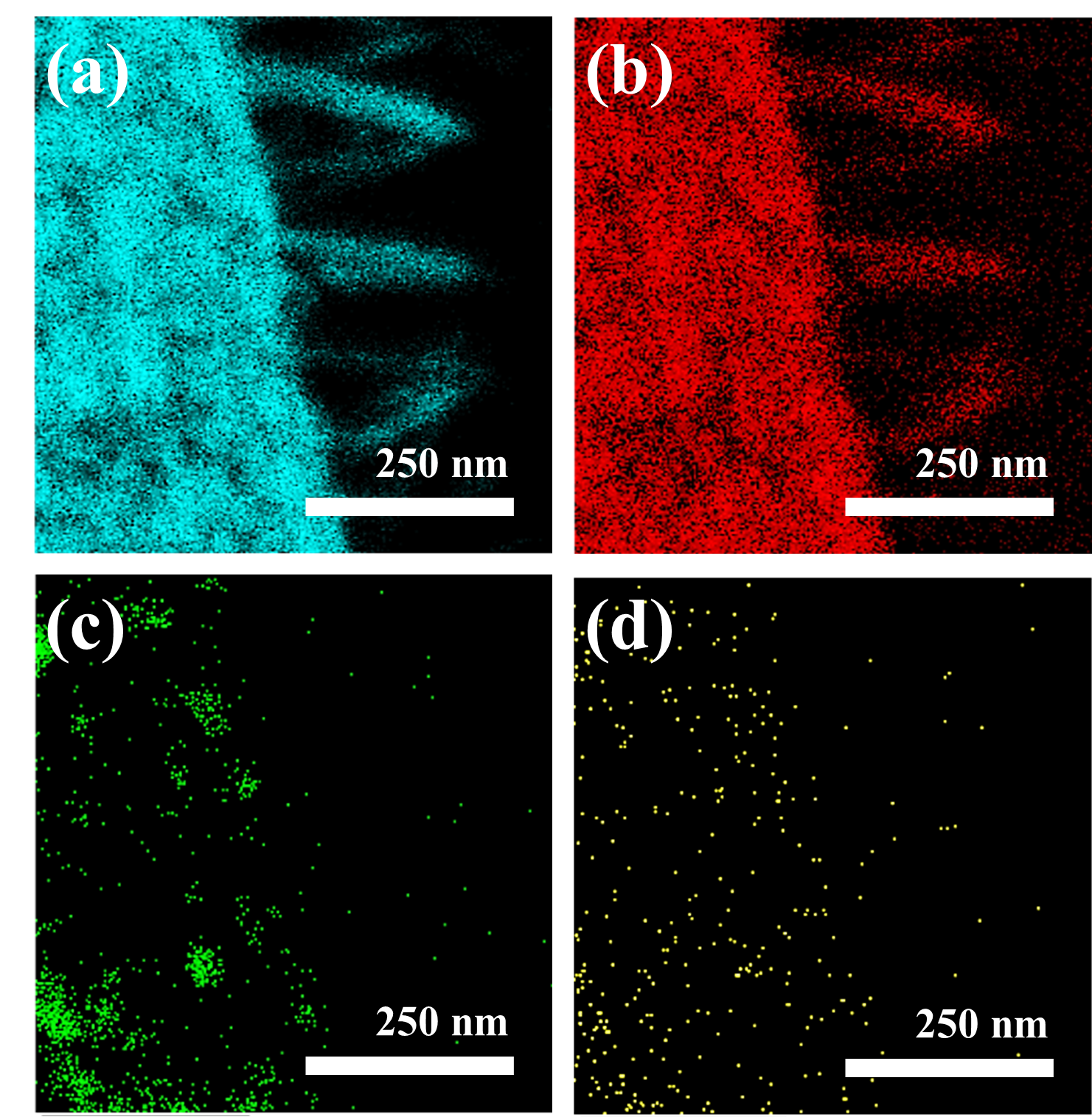


**Figure S1**. EDX elemental mapping images of (a) Zn, (b) O, (c) Sn, and (d) Sb atoms in ZnO NW-loaded 1:9 ATO-ZnO MP Figure 1c.


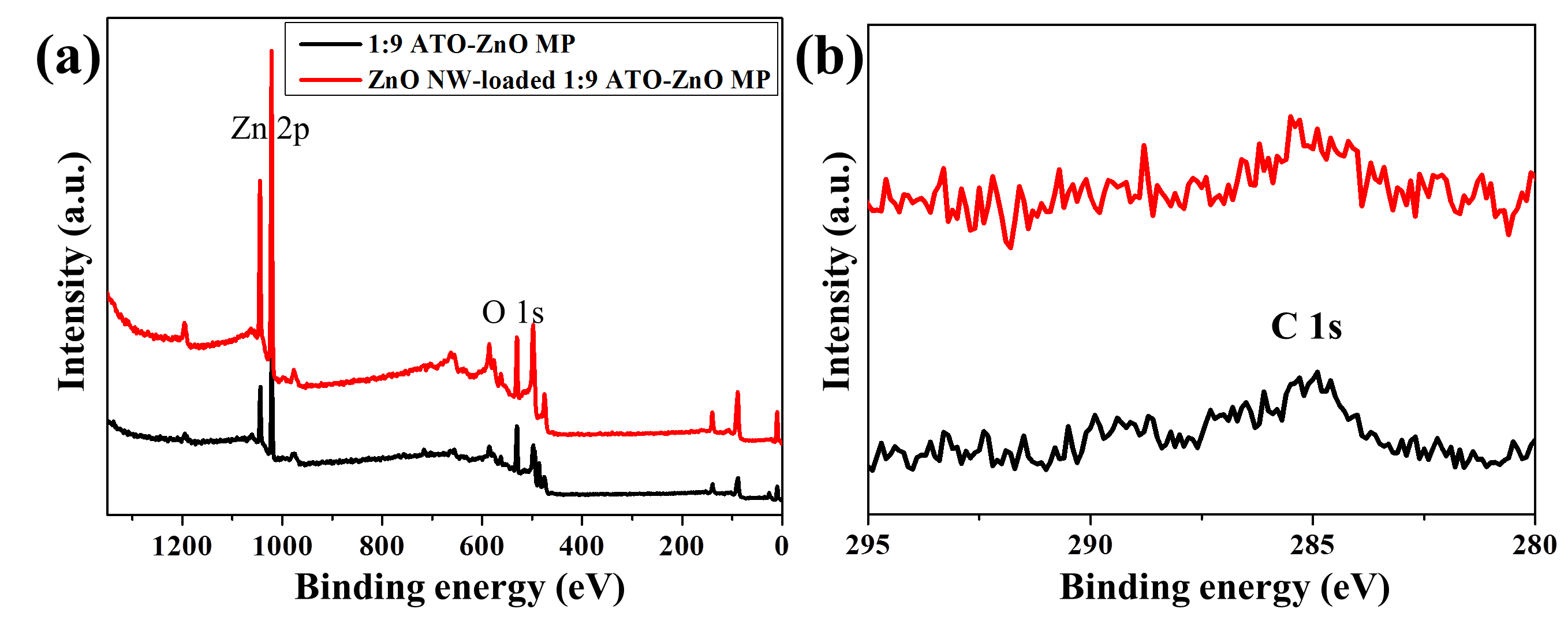


**Figure S2.** XPS spectrum of (a) survey and (b) C1s of 1:9 ATO-ZnO MP and hierarchical ZnO NW-loaded 1:9 ATO-ZnO MP.

**
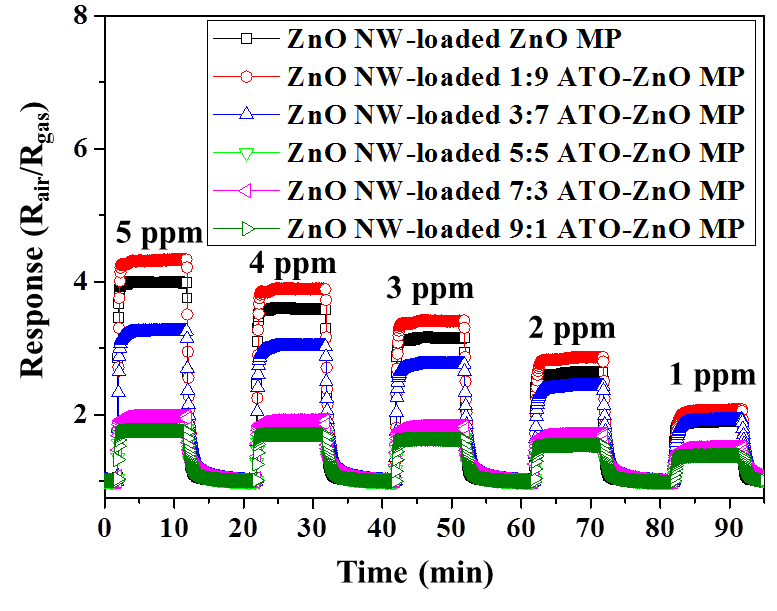
**

**Figure S3.** Dynamic acetone sensing characteristic of ZnO MP and ATO-ZnO MP sensors with 5 different compositions at 400 °C in a concentration range of 1–5 ppm.

**
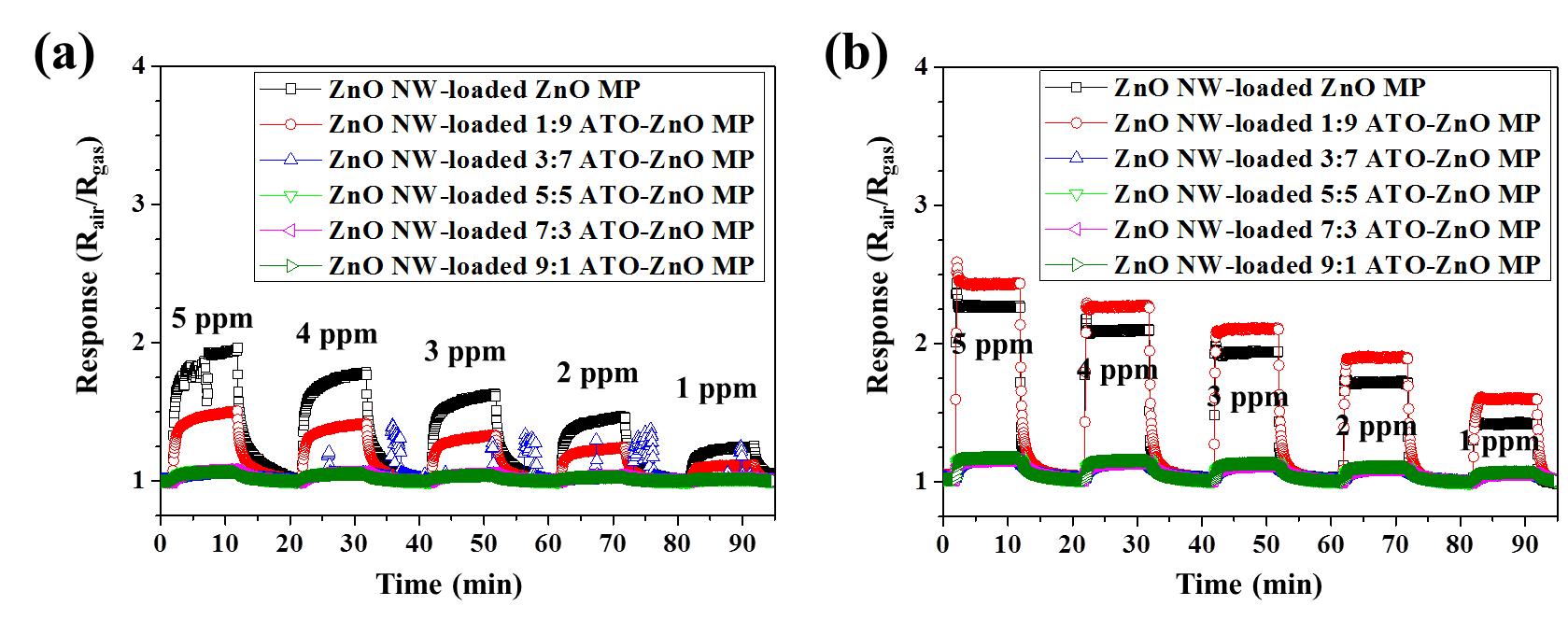
**

**Figure S4.** Dynamic (a) H_2_S- and (b) Toluene-sensing characteristic of ZnO NW-loaded ZnO MP and ZnO NW-loaded ATO-ZnO MP sensors with five different compositions at 400 °C in a concentration range of 1–5 ppm.


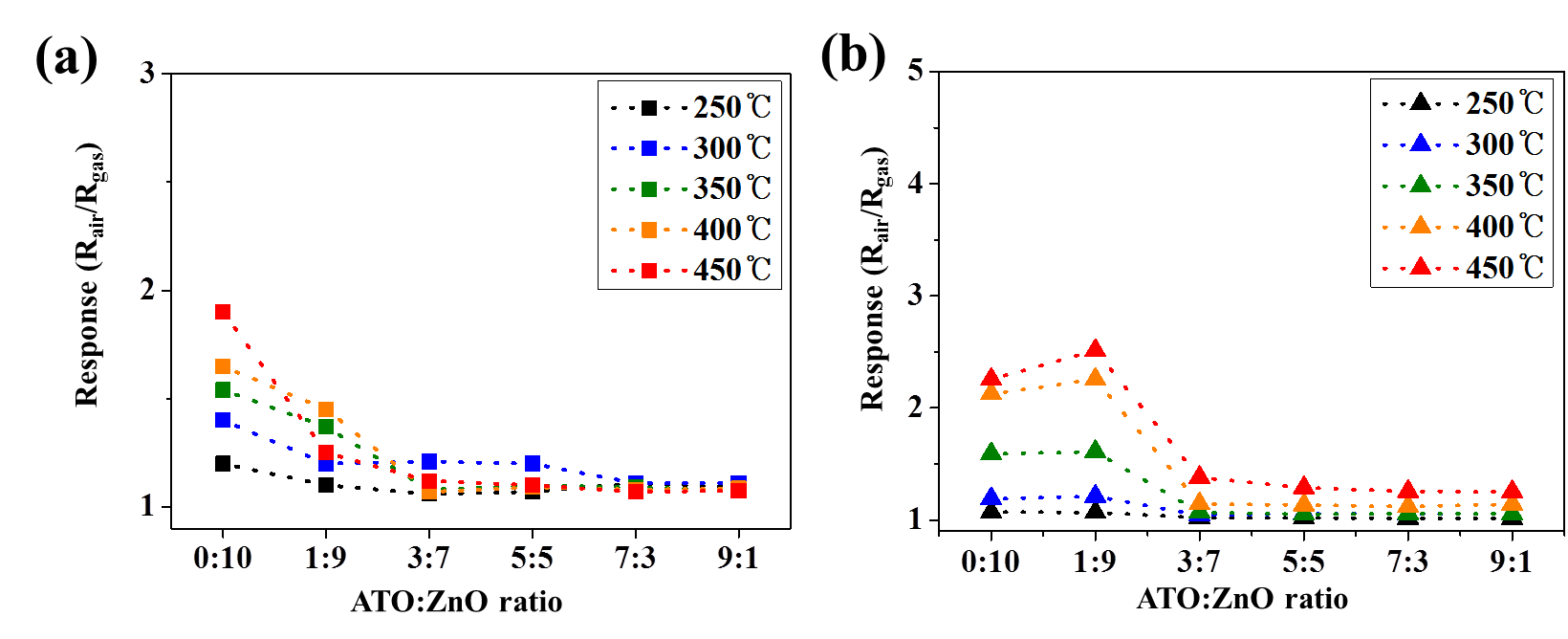


**Figure S5.** Temperature-dependent (a) H_2_S- and (b) Toluene-sensing characteristics of ZnO NW-loaded ZnO MP and ZnO NW-loaded ATO-ZnO MP sensors with five different compositions at 5 ppm in a temperature range of 250–450 °C.


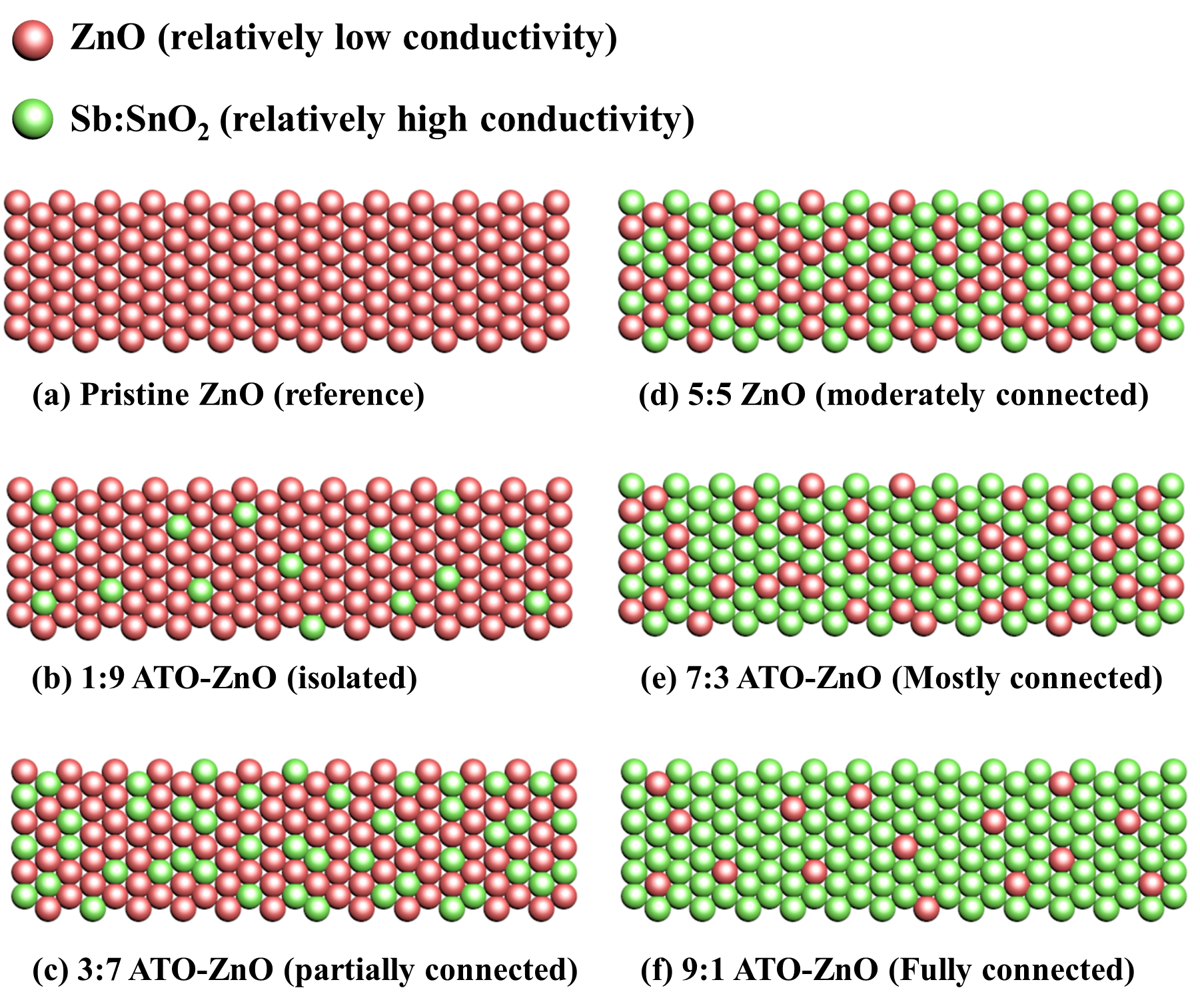


**Figure S6.** Schematic diagram illustrating the relative distribution of ATO and ZnO NPs in a single microstrip line with six different compositional ratios: (a) Pristine ZnO and (b) 1:9, (c) 3:7, (d) 5:5, (e) 7:3, and (f) 9:1 of ATO-ZnO composite.


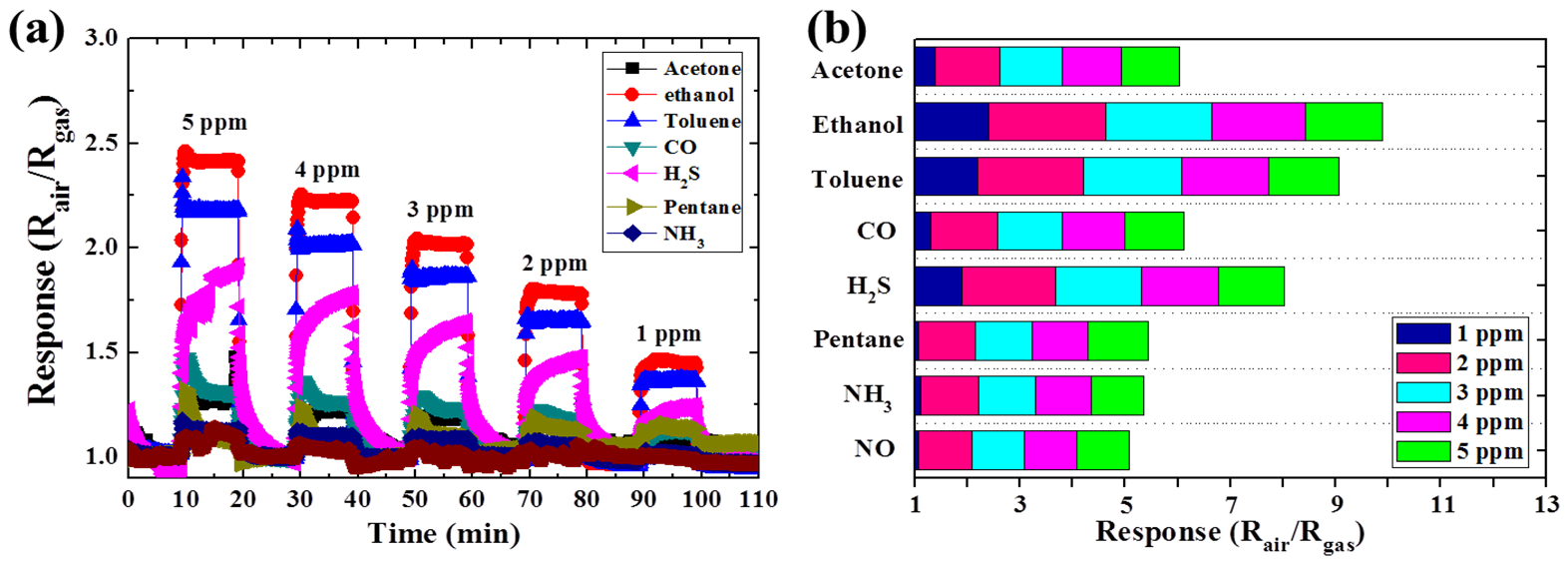


Figure S7. (a) Dynamic response transition of the pristine ZnO NW-loaded ZnO MP toward acetone in the concentration range of 1–5 ppm at 400°C. (b) Selective sensing property of the pristine ZnO NW-loaded ZnO MP toward multiple interfering analytes at 400°C.
